# Indoxyl sulfate induces left ventricular hypertrophy *via* the AhR-FGF23-FGFR4 signaling pathway

**DOI:** 10.3389/fcvm.2023.990422

**Published:** 2023-02-21

**Authors:** Hiroshi Kishimoto, Toshiaki Nakano, Kumiko Torisu, Masanori Tokumoto, Yushi Uchida, Shunsuke Yamada, Masatomo Taniguchi, Takanari Kitazono

**Affiliations:** ^1^Department of Medicine and Clinical Science, Graduate School of Medical Sciences, Kyushu University, Fukuoka, Japan; ^2^Fukuoka Red Cross Hospital, Fukuoka, Japan; ^3^Fukuoka Renal Clinic, Fukuoka, Japan

**Keywords:** indoxyl sulfate, fibroblast growth factor 23, fibroblast growth factor receptor 4, left ventricular hypertrophy, aryl hydrocarbon receptor

## Abstract

**Background:**

Patients with chronic kidney disease (CKD) have a high risk of left ventricular hypertrophy (LVH). Fibroblast growth factor 23 (FGF23) and indoxyl sulfate (IS) are associated with LVH in patients with CKD, but the interactions between these molecules remain unknown. We investigated whether IS contributes to LVH associated with FGF23 in cultured cardiomyocytes and CKD mice.

**Methods and results:**

In cultured rat cardiac myoblast H9c2 cells incubated with IS, mRNA levels of the LVH markers atrial natriuretic factor, brain natriuretic peptide, and β-myosin heavy chain were significantly upregulated. Levels of mRNA of the polypeptide N-acetylgalactosaminyltransferase 3 (GALNT3), which regulates FGF23 O-glycosylation, and FGF23 were also upregulated in H9c2 cells. Intact FGF23 protein expression and fibroblast growth factor receptor 4 (FGFR4) phosphorylation were increased in cell lysates by IS administration. In C57BL/6J mice with heminephrectomy, IS promoted LVH, whereas the inhibition of FGFR4 significantly reduced heart weight and left ventricular wall thickness in IS-treated groups. While there was no significant difference in serum FGF23 concentrations, cardiac FGF23 protein expression was markedly increased in IS-injected mice. GALNT3, hypoxia-inducible factor 1 alpha, and FGF23 protein expression was induced in H9c2 cells by IS treatment and suppressed by the inhibition of Aryl hydrocarbon receptor which is the receptor for IS.

**Conclusion:**

This study suggests that IS increases FGF23 protein expression via an increase in GALNT3 and hypoxia-inducible factor 1 alpha expression, and activates FGF23-FGFR4 signaling in cardiomyocytes, leading to LVH.

## Introduction

Patients with chronic kidney disease (CKD), including those with end-stage kidney disease, have a high risk of cardiovascular disease, which is the major cause of death in these patients (1–5). Traditional cardiovascular risk factors for the general population, such as diabetes mellitus, high blood pressure, and dyslipidemia, are more common in patients with CKD, but cannot entirely explain the increased cardiovascular risk (6). Left ventricular hypertrophy (LVH), which is a typical pathological feature of uremic cardiomyopathy, is an independent risk factor for mortality in patients with CKD (7).

Dysregulated phosphorus metabolism is a common complication of CKD and is associated with adverse cardiovascular and renal outcomes. Fibroblast growth factor 23 (FGF23), which is a hormone secreted by osteocytes, controls the rate of urinary excretion of phosphate and inhibits renal production of 1,25-dihydroxyvitamin D. This process helps to mitigate hyperphosphatemia in patients with kidney disease. Increased circulating FGF23 concentrations are associated with LVH, kidney disease progression, and mortality in patients with dialysis, predialysis CKD, or non-CKD, independent of risk factors, such as high phosphate and parathyroid hormone concentrations (8–14).

Indoxyl sulfate (IS) is an important uremic solute, which is derived from tryptophan in dietary protein. IS is converted in the liver and excreted into the urine by proximal tubular secretion *via* organic anion transporters (15). Serum IS greatly and progressively increases with increasing CKD stages, and is a predictor of overall and cardiovascular mortality. In addition, the serum IS concentration is independently associated with LVH in patients with CKD (16).

FGF23 and IS are associated with LVH in patients with CKD, but the interactions between these molecules remain unknown. Therefore, we examined whether IS is associated with FGF23 and induces LVH *in vivo* and *in vitro*.

## Materials and methods

### Cell culture

Rat cardiac myoblast (H9c2, 2-1) cells were acquired from the American Type Culture Collection (Manassas, VA) and cultivated in Dulbecco’s Modified Eagle Medium (GIBCO, Dublin, Ireland) containing 10% fetal bovine serum (HyClone; GE Healthcare, Bucks, UK), 100 U/mL penicillin, and 100 mg/mL streptomycin (Life Technologies, Carlsbad, CA), in a humidified atmosphere with 5% CO_2_ at 37°C. The cells were cultured until 70–80% confluence and were then serum deprived for 24 h before each experiment. The cells were incubated with 0, 0.25, or 1.0 mM IS or 0, 50, or 100 ng/mL recombinant mouse FGF23 (2629-FG; R&D Systems, Minneapolis, MN) diluted in normal saline (NS) and collected after 24 and 72 h for real-time reverse transcription-polymerase chain reaction and western blotting, respectively. To perform small interfering RNA (siRNA) knockdown, the cells were transfected with On-TARGETplus SMARTpool siRNA [non-targeting control, aryl hydrocarbon receptor (AhR), and FGF23; Horizon Discovery, Cambridge, UK] using Dharmafect 1 reagent (Horizon Discovery) in accordance with the manufacturer’s instructions.

### Animal experiments and ethics statement

All animal experiments were conducted in accordance with the animal use protocols approved by the Committee on Ethics of Animal Experimentation at Kyushu University Graduate School of Medical Sciences (Approval number: A19-274-0). We used C57BL/6Jcl mice (CLEA Japan, Inc., Tokyo, Japan). They were maintained in an air-conditioned specific pathogen-free room at 21°C and 65% humidity, with a 12:12-h light and dark cycle (lights on at 8:00 a.m., off at 8:00 p.m.) with free access to chow and water. Experiments were reported according to the ARRIVE guidelines.

### Experimental procedures for LVH in mice

To induce LVH, 8-week-old male mice were fed a high phosphorus diet, which contained modified AIN-93G, lactose 20.0%, sucrose 2.023%, β-corn starch 20.3486%, α-corn starch 7.0%, CaCO_3_ 0.55%, Ca(H_2_PO_4_)_2_ 5.05%, and phosphate 1.5g/100g (Oriental Yeast, Tokyo, Japan) 1 day after left heminephrectomy (removal of the whole left kidney). This diet was provided for the induction of FGF23 and was administered with a continuous subcutaneous dose of 100 mg/kg IS (I3875; Sigma-Aldrich, St. Louis, MO) or 28 μl/day of NS (Otsuka Pharmaceutical Factory, Tokushima, Japan) for 4 weeks using a micro-osmotic pump (2002-0000296; Alzet, Cupertino, CA). Half of the mice were treated with a continuous intraperitoneal dose of 7.5 mg/kg H3B-6527 (H3B), which is an FGFR4 inhibitor (S8675; Selleck Biotech, Tokyo, Japan), or 3.6 μl/day of NS using a micro-osmotic pump (1004-0009922; Alzet) for 4 weeks. The 2,002-pumps were replaced biweekly. To examine the effect of IS administration on cardiac hypertrophy and fibrosis, the mice were divided into the five following groups: sham; control; IS + NS; NS + H3B; and IS + H3B. Sham mice were treated with anesthesia, skin incision, and laparotomy, but did not have pumps inserted and were fed a normal phosphorus diet (CRF-1LID10; Oriental Yeast, Tokyo, Japan). The mice were operated on day 0, observed for 4 weeks, and euthanized on day 28. The mice were anesthetized intraperitoneally with medetomidine hydrochloride (0.3 mg/kg body weight; Wako, Osaka, Japan), midazolam (4 mg/kg body weight, Sandoz, Tokyo, Japan), and butorphanol tartrate (5 mg/kg body weight, Wako). The body temperature of the mice was maintained at 37°C during the whole procedure.

### Sample collection

The mice were euthanized on day 28. Blood samples were collected from the inferior vena cava. Serum was separated by centrifugation at 3,000 × *g* for 10 min, aliquoted for later analysis, and stored at −80°C. Immediately after blood collection, 50 mL ice-cold phosphate-buffered saline (14249-24; Nacalai Tesque Inc., Kyoto, Japan) (pH 7.4) was slowly perfused to harvest the heart and kidney. We separated the right kidney in half cross-sectionally for obtaining coronal sections. One half of the kidney and most of the heart were immersed in 10% formaldehyde neutral buffer solution (37152-51; Nacalai Tesque Inc.) for histological analysis. The other half of the kidney and the apex of the heart (one quarter of the heart) were snap-frozen in liquid nitrogen and stored at −80°C for protein and RNA analysis.

### Heart histology

The hearts of mice were immersed in 10% formaldehyde neutral buffer solution for at least 48 h before being sectioned. The hearts were cut horizontally into four equal sections. The thickest wall of the left ventricle from the four sections was evaluated. Sections from each sample were subjected to hematoxylin–eosin staining using standard methods. Histological images were captured by light microscopy (Eclipse E800 microscope; Nikon, Tokyo, Japan).

### RNA extraction and quantitative real-time polymerase chain reaction

Total RNA was extracted from H9c2 cells using the MAXWELL^®^16 LEV simply RNA tissue Kit (Promega, Madison, WI) and the MAXWELL^®^ 16 instrument (Promega) in accordance with the manufacturer’s instructions. Complementary DNA was synthesized from 1 μg of total RNA with the PrimeScript RT Reagent Kit (Takara Bio Inc., Shiga, Japan). Real-time polymerase chain reaction (PCR) was performed using SYBR Premix Ex Taq™ (Takara Bio Inc.) and the Applied Biosystems 7,500 Real Time PCR System (Applied Biosystems, Foster, CA). Rat Glyceraldehyde 3-phosphate dehydrogenase (*GAPDH*) was amplified as an internal control. Expression analysis was performed by the Delta-Delta Ct method using 7500 Software v2.3 (Applied Biosystems). The primers used for these experiments were as follows: rat atrial natriuretic factor (*ANF*), forward 5′-ATGGGCTCCTTCTCCATCAC-3′ and reverse 5′-TTCATCGGTATGCTCGCTCA-3′; rat brain natriuretic peptide (*BNP*), forward 5′-TGGGAAGTCCTAGCCAGTCT-3′ and reverse 5′-GATCCGGTCTATCTTCTGCC-3′; rat β-myosin heavy chain (*beta MHC*), forward 5′-CTAGGAGGCGGAGGAACAG-3′ and reverse 5′-CTTGGCGCCAATGTCACG-3′; rat alpha smooth muscle actin (*alpha SMA*), forward 5′-GACACCAGGGAGTGATGGTT-3′ and reverse 5′-GTTAGCAAGGTCCGATGCTC-3′; rat collagen I, forward 5′-TGCCGTGACCTCAAGATGTG-3′ and reverse 5′-CACAAGCGTGCTGTAGGTGA-3′; rat *FGF23*, forward 5′-GCAACATTTTTGGATCGTATCA-3′ and reverse 5′-GATGCTT CGGTGACAGGTAGA-3′; rat N-acetylgalactosaminyltransferase 3 (*GALNT3*), forward 5′-GTTGCTAGGAGCAACAGTCGCA-3′ and reverse 5′-AGTTCACCGTGGTAGTATTGTAGT-3′; and rat *GAPDH*, forward 5′-GGCACAGTCAAGGCTGAGAATG-3′ and reverse 5′-ATGGTGGTGAAGACGCCAGTA-3′.

### Western blot analysis

To perform western blot analysis, H9c2 cells were harvested with cell lysis buffer (Mammalian Protein Extraction Reagent, 78501; Pierce Thermo Scientific, Tokyo, Japan) containing protease inhibitors (#04080-11; Nacalai Tesque Inc.) and phosphatase inhibitors (#07575-51; Nacalai Tesque Inc.) on ice for 15 min. The supernatants of protein lysates were collected after 10 min of centrifugation at 10,000 × *g*. The protein concentrations of cell lysates were determined using a bicinchoninic acid (BCA) protein assay kit (Thermo Fisher Scientific). The samples (5 μg) were separated on 5–20% sodium dodecyl sulfate-polyacrylamide gels (#2331830; Atto, Tokyo, Japan) and transferred onto polyvinylidene difluoride membranes (BioRad, Hercules, CA) using Trans-Blot Turbo (BioRad). After being blocked with Blocking One (#03953-95; Nacalai Tesque Inc.) for 30 min at room temperature, the membranes were washed in Tris-buffered saline containing 0.1% Tween 20 (polyoxyethylene sorbitan monolaurate, 35624-15; Nacalai Tesque Inc.) three times for 10 min and incubated with primary antibodies at 4°C overnight. The following antibodies were used as primary antibodies: monoclonal anti-rat FGF23 antibody (1:500, MAB2629; R&D Systems); polyclonal anti-goat FGF23 antibody (1:1000, ab123502; Abcam, Cambridge, UK); polyclonal anti-rabbit FGFR4 antibody (1:1000, ab119378; Abcam); polyclonal anti-rabbit FGFR4 (phospho Y642; pFGFR4) antibody (1:1000, ab192589; Abcam); polyclonal anti-rabbit furin antibody (1:1000, PA1-062; Thermo Fisher Scientific); polyclonal anti-rabbit hypoxia-inducible factor 1 alpha (HIF1α) antibody (1:1000, NB100-134; Novus Biologicals, Centennial, CO); polyclonal anti-rabbit polypeptide GALNT3 antibody (1:1000, SAB2106736; Sigma-Aldrich); and anti-rabbit α/β tubulin (1:1000, CST#2148; Cell Signaling Technology, Danvers, MA). After being washed in Tris-buffered saline containing 0.1% Tween 20 three times, the membranes were incubated with the following horseradish peroxidase-conjugated secondary antibodies: donkey anti-rabbit IgG antibody (1:5000, NA934; GE Healthcare, Bucks, UK) and goat anti-rat IgG antibody (1:10,000, NA935; GE Healthcare) for 1 h. The bands were detected by the enhanced chemiluminescent method (ECL prime; GE Healthcare or Chemi-Lumi One Ultra; Nacalai Tesque Inc.), captured using a chemiluminescence imaging system (AE-9300 Ez-capture MG; Atto), and analyzed with ImageJ Software (National Institutes of Health, Bethesda, MD).

### Statistical analysis

Data were analyzed using the JMP 14.0 software program (SAS Institute, Tokyo, Japan). Continuous variables are expressed as the mean ± standard error. Differences between two groups were compared by Student’s *t*-test. Differences among groups were compared by one-way analysis of variance followed by Tukey’s honest significant difference tests. *P* < 0.05 was considered statistically significant for all tests.

## Results

### FGF23 Promotes hypertrophic and pro-fibrotic signaling in cultured cardiomyocytes

**FIGURE 1:**
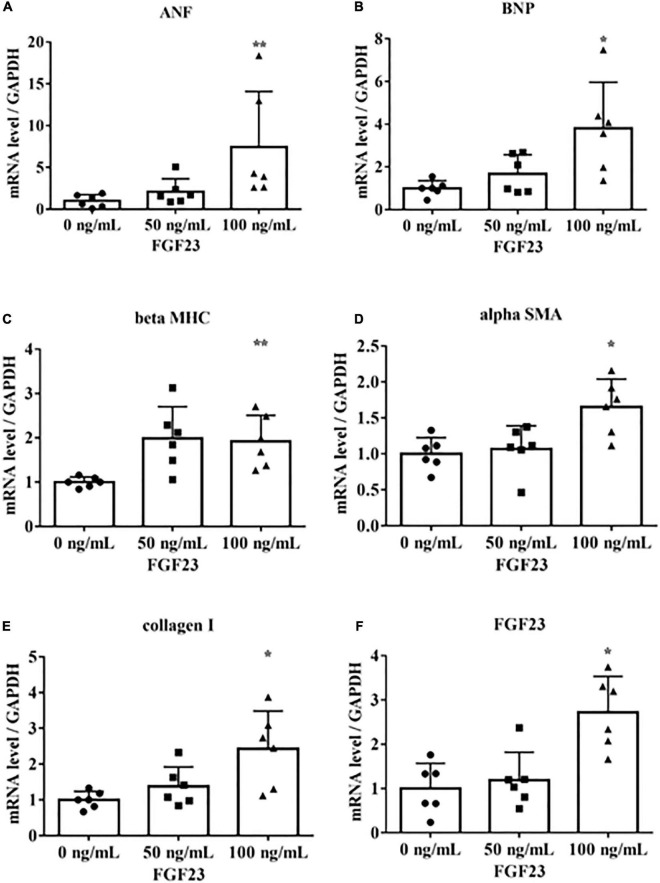
Fibroblast growth factor 23 increases mRNA levels of markers of hypertrophy and fibrosis. Rat cardiac myoblast cells (H9c2) were cultured with 0, 50, or 100 ng/mL fibroblast growth factor 3 (FGF23) for 24 h, and mRNA expression levels were analyzed by real-time PCR. **(A)** Atrial natriuretic factor (ANF) (*n* = 6). **P < 0.05 versus 0 ng/mL. **(B)** Brain natriuretic peptide (BNP) (*n* = 6). *P < 0.01 versus 0 ng/mL. **(C)** β-myosin heavy chain (*beta MHC*) (*n* = 6). **P < 0.05 versus 0 ng/mL. **(D)** Alpha smooth muscle actin (*alpha SMA*) (*n* = 6). *P < 0.01 versus 0 ng/mL. **(E)** Collagen I (*n* = 6). *P < 0.01 versus 0 ng/mL. **(F)** *FGF23* (*n* = 6). *P < 0.01 versus 0 ng/mL. Data were analyzed by one-way analysis of variance.

To determine if FGF23 is associated with hypertrophic signaling in cultured rat cardiac myoblast cells (H9c2), we incubated H9c2 cells with 0, 50, or 100 ng/mL recombinant mouse FGF23. These concentrations were chosen on the basis of previous reports (17–19). The cells were collected after 24 h for real-time PCR. The mRNA levels of *FGF23*, the LVH markers *ANF, BNP*, and *beta MHC*, and the fibrotic markers *alpha SMA* and collagen I were upregulated (Figure 1).

### IS upregulates hypertrophic and pro-fibrotic signaling and increases FGF23 expression in cultured cardiomyocytes

**FIGURE 2:**
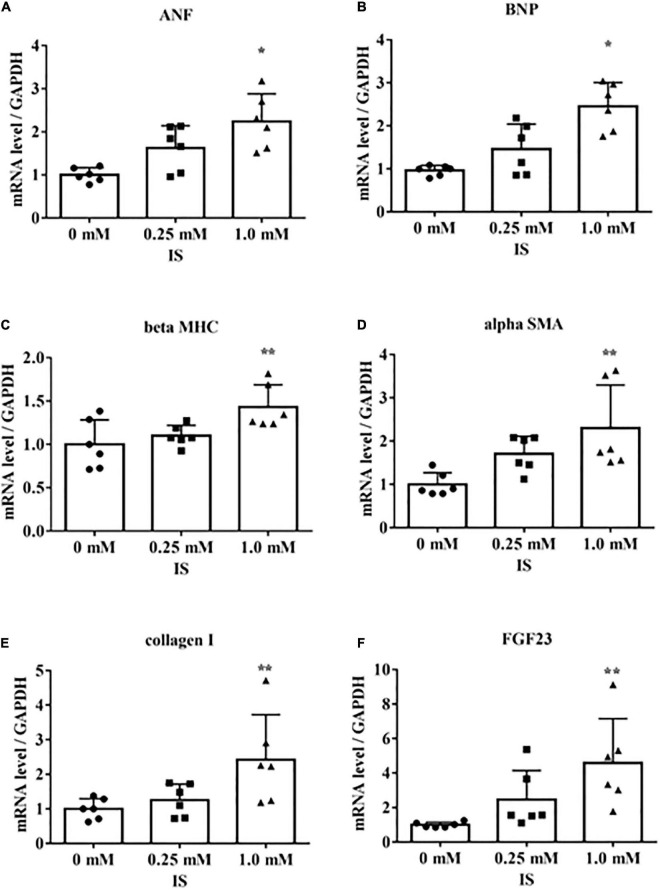
Indoxyl sulfate increases mRNA levels of markers of hypertrophy and fibrosis. H9c2 cells were cultured with 0, 0.25, or 1.0 mM indoxyl sulfate (IS) for 24 h, and mRNA expression levels were analyzed by real-time PCR. **(A)** Atrial natriuretic factor (*ANF*) (*n* = 6). *P < 0.01 versus 0 mM. **(B)** Brain natriuretic peptide (BNP) (*n* = 6). *P < 0.01 versus 0 mM. **(C)** β-myosin heavy chain (*beta MHC*) (*n* = 6). **P < 0.05 versus 0 mM. **(D)** Alpha smooth muscle actin (*alpha SMA*) (*n* = 6). **P < 0.05 versus 0 mM. **(E)** Collagen I (*n* = 6). **P < 0.05 versus 0 mM. **(F)** *FGF23* (*n* = 6). **P < 0.05 versus 0 mM. Data were analyzed by one-way analysis of variance.

We determined the underlying mechanisms by which IS induces cardiomyocyte hypertrophy by treating H9c2 cells with IS. The H9c2 cells were cultured with 1 mM IS for 24 h, which has been reported as a clinically relevant concentration of IS in severe CKD (20–23). The mRNA levels of *beta MHC, BNP, alpha SMA*, collagen I, and *FGF23* were upregulated (Figure 2). We also examined whether IS affects the cell size in cultured cardiomyocytes. We found that IS induced hypertrophy in H9c2 cells (Supplementary Figure 1). IS is known as a protein-bound toxin (20). Therefore, we examined whether IS induces hypertrophic and pro-fibrotic signaling in H9c2 cells with 4% albumin-containing medium as previously reported (23). IS induced hypertrophic and pro-fibrotic signaling in H9c2 cells in the 4% albumin-containing medium (Supplementary Figure 2).

**FIGURE 3:**
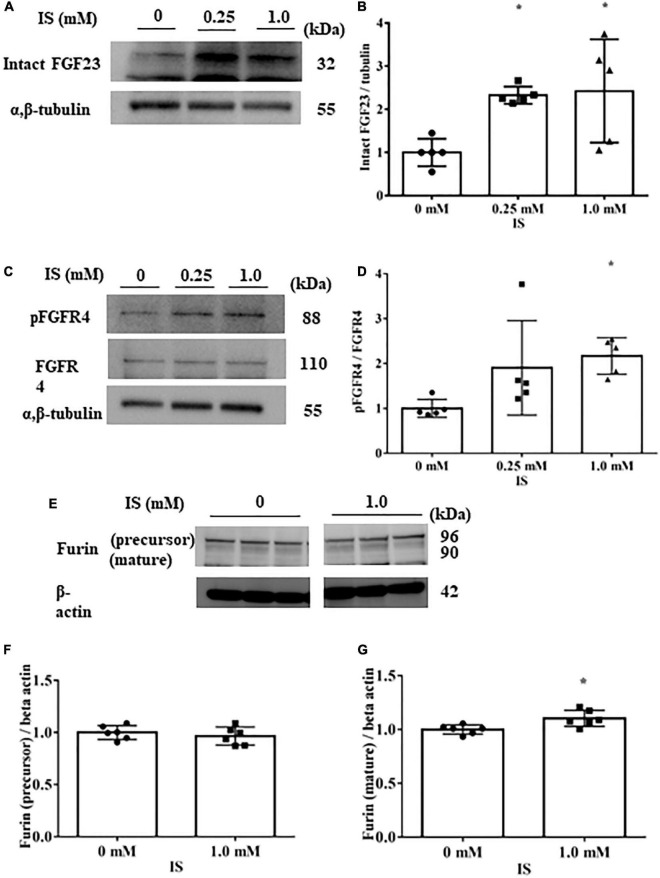
Indoxyl sulfate increases fibroblast growth factor 23 protein expression and fibroblast growth factor receptor 4 phosphorylation. **(A,B)** Western blotting of fibroblast growth factor 3 (FGF23) protein expression in H9c2 cells. H9c2 cells were cultured with 0, 0.25, or 1.0 mM indoxyl sulfate (IS) for 72 h. Alpha, beta-tubulin protein expression was examined as an internal control (*n* = 5). *P < 0.05 versus IS 0 mM. **(C,D)** Western blotting of fibroblast growth factor receptor 4 (FGFR4) protein expression and FGFR4 phosphorylation in H9c2 cells. H9c2 cells were cultured with 0, 0.25, or 1.0 mM IS for 72 h. Alpha, beta-tubulin protein expression was examined as an internal control (*n* = 5). *P < 0.05 versus IS 0 mM. **(E–G)** Western blotting of furin protein in H9c2 cells. H9c2 cells were cultured with 0 or 1.0 mM IS for 72 h. The images are from different parts of the same gel. Beta actin protein expression was examined as an internal control (*n* = 6). Furin stained as two bands, furin precursor (96 KDa) and mature (90 KDa) forms. *P < 0.05 versus IS 0 mM. Data were analyzed by one-way analysis of variance.

In H9c2 cells cultured with 1 mM IS for 72 h, FGF23 protein expression and FGFR4 phosphorylation were elevated in cell lysates (Figures 3A–D). This FGFR4 phosphorylation by IS also occurred even in the 4% albumin-containing medium (Supplementary Figure 3). Furin, which is a protein that cleaves intact FGF23, was not downregulated by IS (Figures 3E, F).

These data suggest that IS increases FGF23 protein expression and FGFR4 phosphorylation in cardiomyocytes, and induces hypertrophic and pro-fibrotic signaling in cardiomyocytes.

### IS induces LVH and FGFR4 inhibition reduces this effect

**FIGURE 4:**
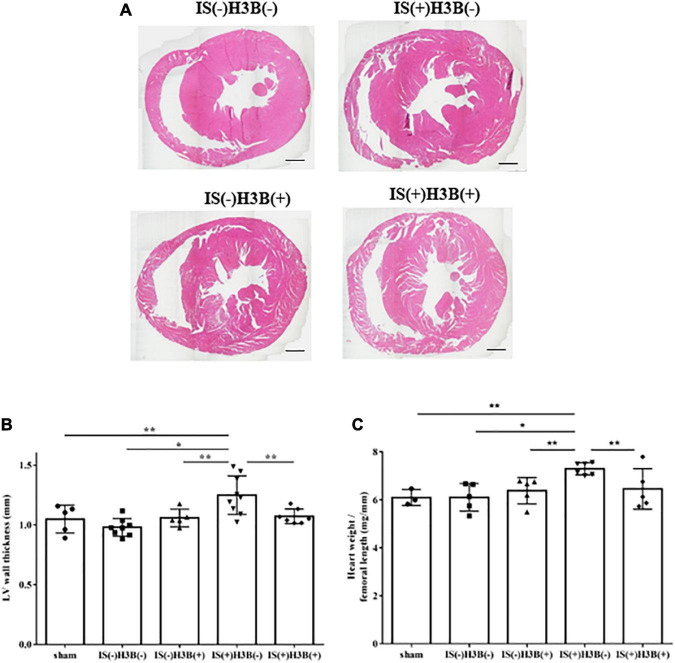
Indoxyl sulfate induces LVH in mice with CKD. **(A)** Mouse hearts were cut horizontally and stained with hematoxylin–eosin. Representative images are shown. Scale bar = 500 μm. **(B)** After hematoxylin–eosin staining, we measured the LV wall thickness for each sample (*n* = 3–6). *P < 0.01, **P < 0.05. **(C)** The relative heart weight was markedly increased in indoxyl sulfate (IS)-treated mice and this increase was reduced by FGFR4 inhibition (*n* = 3–6). *P < 0.01, **P < 0.05. Data were analyzed by one-way analysis of variance.

**FIGURE 5:**
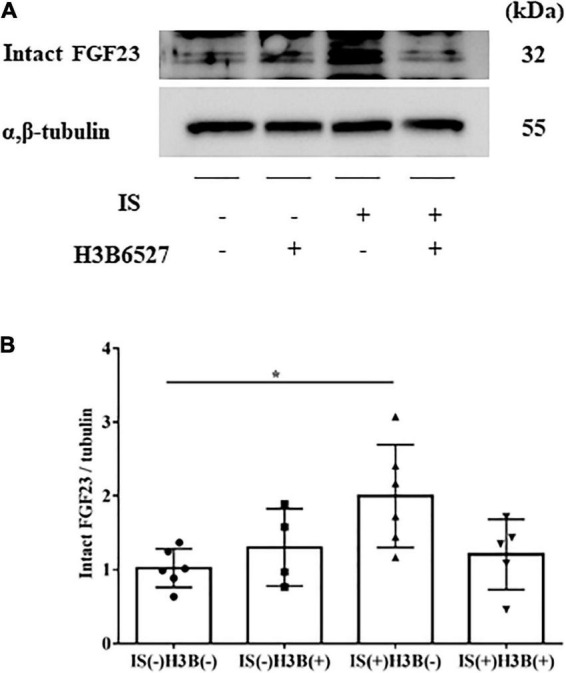
**(A,B)** IS increases intact FGF23 expression in the heart. The expression of intact FGF23 in the heart was significantly increased after IS treatment. This effect was abrogated by FGFR4 inhibition as shown by Western blotting (*n* = 3–6). Data were analyzed by one-way analysis of variance. *P < 0.05.

**FIGURE 6:**
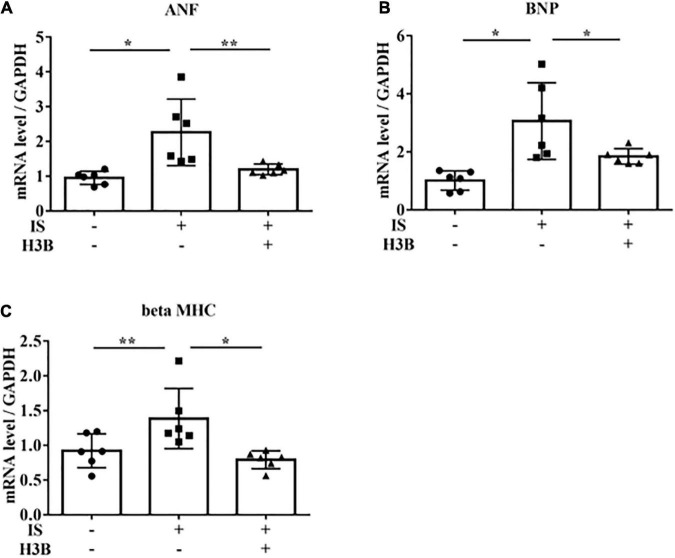
IS increases hypertrophic markers *via* FGFR4 *in vitro*. H9c2 cells were cultured with 0 or 1 mM IS and 0 or 10 μM H3B-6527 (FGFR4 inhibitor) for 6 h. **(A)** *ANF* mRNA levels were significantly increased by IS and downregulated with H3B-6527 (*n* = 6). *P < 0.01, **P < 0.05. **(B)** *BNP* mRNA levels were significantly increased by IS and downregulated with H3B-6527 (*n* = 6). *P < 0.01. **(C)** *beta MHC* mRNA levels were significantly increased by IS and downregulated with H3B-6527 (*n* = 6). *P < 0.01, **P < 0.05. Data were analyzed by one-way analysis of variance.

In our mild CKD mouse model, we observed a heavier heart weight and greater left ventricular wall thickness in IS + NS mice than in the other mice (Figures 4A–C). Body weight, blood pressure, creatinine concentrations, calcium concentrations, and phosphate concentrations were not significantly different among the groups (Table 1). Serum IS concentrations tended to be increased in the IS-treated groups (Table 1). Inhibition of FGFR4 reduced heart weight and left ventricular wall thickness in the IS-treated groups (Figures 4A–C). Although serum FGF23 concentrations were not significantly different among the experimental groups (Table 1), FGF23 protein expression in the heart was markedly increased in IS-injected mice (Figures 5A, B). To examine the relationship between IS and FGFR4, we treated H9c2 cells with IS with or without an FGFR4 inhibitor for 24 h (Figure 6). IS upregulated mRNA expression levels of the hypertrophic markers *ANF, BNP*, and *beta MHC*, and FGFR4 inhibition reduced their expression.

**TABLE 1:** Body weight, blood pressure, and laboratory data from animal experiments.

	Sham	IS(-)H3B(-)	IS(-)H3B(+)	IS(+)H3B(-)	IS(+)H3B(+)	*p*
Body wight (g)	24.76 ± 1.25	22.41 ± 1.91	23.07 ± 0.74	24.18 ± 0.98	23.44 ± 1.91	0.181
Blood pressure (mmHg)	121.03 ± 15.5	135.8 ± 14.7	127.9 ± 19.0	116.1 ± 13.4	133.3 ± 13.8	0.243
Cre (mg/dL)	0.55 ± 0.38	0.22 ± 0.17	0.31 ± 0.22	0.18 ± 0.03	0.31 ± 0.24	0.189
Ca (mg/dL)	10.20 ± 1.04	9.46 ± 0.59	9.76 ± 0.47	10.28 ± 0.78	9.88 ± 0.63	0.359
P (mg/dL)	13.17 ± 1.32	12.96 ± 1.95	16.28 ± 3.29	17.47 ± 5.56	17.24 ± 1.77	0.161
Intact FGF23 (pg/mL)	104.4 ± 88.5	857.8 ± 339.5*	728.0 ± 238.2*	832.7 ± 257.6*	658.7 ± 175.1*	0.0047
Indoxyl sulfate (μg/dL)	3.48 ± 0.99	2.96 ± 1.13	6.51 ± 0.99	11.81 ± 1.11**	9.15 ± 1.35	0.0072

Data are presented as the mean ± standard error. IS, indoxyl sulfate; H3B, H3B-6527; Cre, creatinine; Ca, calcium; P, phosphorus. Comparisons of groups were analyzed with one-way analysis of variance followed by Tukey’s test. *P < 0.01 versus the sham group. **P < 0.05 versus the sham group and the IS(-)H3B(-) group.

### IS stimulates AhR and the FGF23-FGFR4 signaling pathway

**FIGURE 7:**
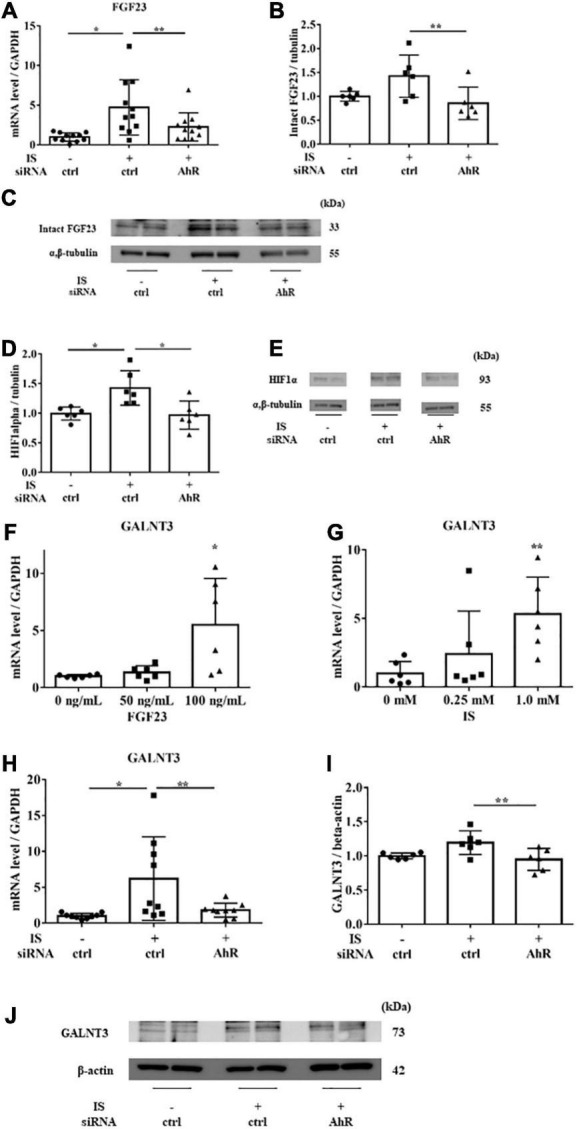
IS induces FGF23 *via* AhR *in vitro*. H9c2 cells were cultured with 0 or 1 mM IS for 6 h (for mRNA) or 48 h (for protein) after pretreatment with AhR siRNA or control siRNA (ctrl). **(A)** *FGF23* mRNA levels were significantly increased by IS and downregulated with AhR siRNA (*n* = 11). *P < 0.01, **P < 0.05. **(B,C)** Western blotting of FGF23 protein expression in H9c2 cells. Alpha, beta-tubulin protein expression was examined as an internal control (*n* = 6). *P < 0.01, **P < 0.05. **(D,E)** Western blotting of HIF1α protein expression in H9c2 cells. The images are different parts of the same gel. Alpha, beta-tubulin protein expression was used as an internal control (*n* = 6). *P < 0.01, **P < 0.05. **(F)** H9c2 cells were cultured with 0, 50, or 100 ng/mL FGF23 for 24 h, and mRNA expression levels were analyzed by real-time PCR. Levels of polypeptide N-acetylgalactosaminyltransferase 3 (*GALNT3*) mRNA (*n* = 6). *P < 0.01, **P < 0.05. **(G)** H9c2 cells were cultured with 0, 0.25, or 1.0 mM IS for 24 h, and mRNA expression levels were analyzed by real-time PCR. Levels of *GALNT3* mRNA (*n* = 6). *P < 0.01, **P < 0.05. **(H)** Levels of *GALNT3* mRNA (*n* = 9). *P < 0.01, **P < 0.05. **(I,J)** Western blotting of GALNT3 protein expression in H9c2 cells. Alpha, beta-tubulin protein expression was used as an internal control (*n* = 6). *P < 0.01, **P < 0.05. Data were analyzed by one-way analysis of variance.

To examine the relationship between IS and FGF23, we suppressed AhR, which is the receptor of IS, by siRNA in H9c2 cells and then treated cells with 1 mM IS for 6 h (for mRNA) or 48 h (for protein expression). The increase in *FGF23* expression in IS-treated myocytes was suppressed by AhR siRNA (Figures 7A–C). HIF1α, which is a hypoxic stress marker that regulates FGF23 production (24), was upregulated by IS. This effect was suppressed by AhR siRNA (Figures 7D, E). GALNT3, which regulates FGF23 O-glycosylation (25), was also upregulated by FGF23 and IS (Figures 7F, G). The increased expression of GALNT3 in IS-treated myocytes was suppressed by AhR siRNA (Figures 7H–J). These data suggest that IS affects FGF23 processing by upregulating GALNT3 and by an inflammatory effect.

### The increase in hypertrophic and pro-fibrotic signaling by IS depends on FGF23 in cultured cardiomyocytes

**FIGURE 8:**
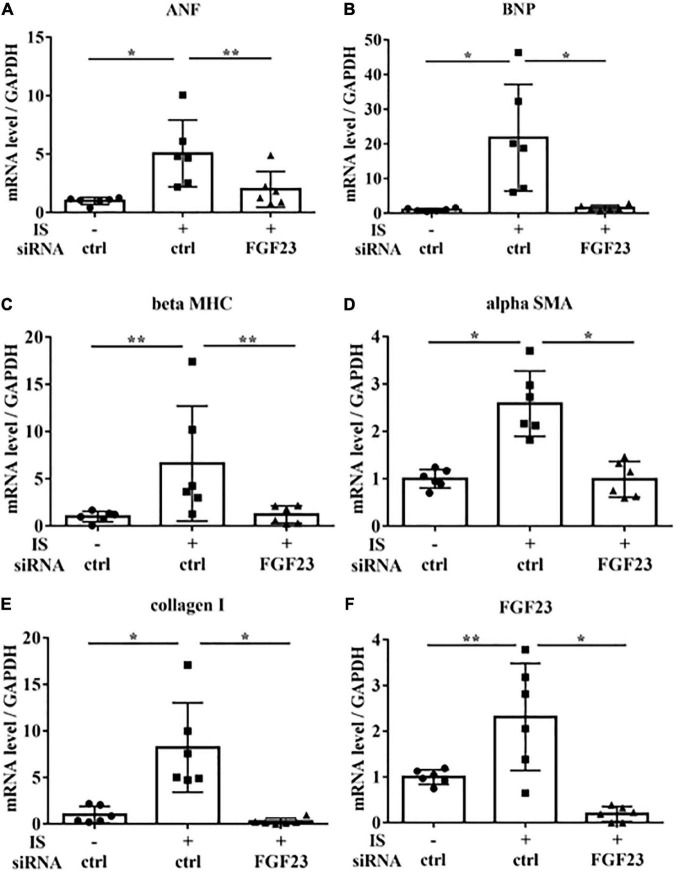
IS increases hypertrophic markers *via* FGF23 *in vitro*. H9c2 cells were cultured with 0 or 1 mM IS for 24 h after pretreatment with FGF23 siRNA or control siRNA (ctrl), and mRNA expression levels were analyzed by real-time PCR. **(A)** *ANF*, **(B)** *BNP*, **(C)** *beta MHC*, **(D)** *alpha SMA*, **(E)** collagen I, and **(F)** *FGF23* mRNA levels were significantly increased by IS and downregulated with FGF23 siRNA (*n* = 6). Data were analyzed by one-way analysis of variance. *P < 0.01, **P < 0.05.

**FIGURE 9:**
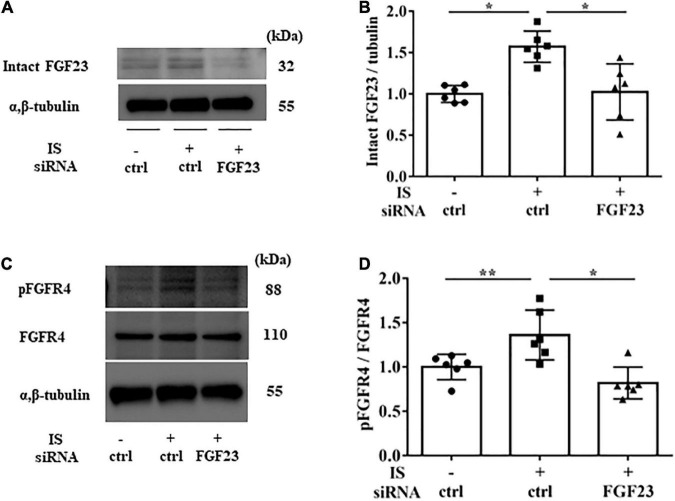
IS increases FGFR4 *via* FGF23 *in vitro*. H9c2 cells were cultured with 0 or 1 mM IS for 48 h after pretreatment with FGF23 siRNA or control siRNA (ctrl). **(A,B)** Western blotting of FGF23 protein expression in H9c2 cells. **(C,D)** Western blotting of FGFR4 protein expression and FGFR4 phosphorylation in H9c2 cells. Alpha, beta-tubulin protein expression was examined as an internal control (*n* = 6). Data were analyzed by one-way analysis of variance. *P < 0.01, **P < 0.05.

To examine the relationship between IS and FGF23, we suppressed FGF23 by siRNA in H9c2 cells and then treated cells with 1 mM IS for 24 h (for mRNA) or 48 h (for protein expression) (Figures 8F, 9A). The increase in mRNA levels of *ANF, beta MHC, BNP, alpha SMA*, collagen I, and *FGF23* was suppressed by FGF23 siRNA (Figures 8A–F). The increase in FGF23 and FGFR4 phosphorylation in IS-treated myocytes was suppressed by FGF23 siRNA (Figures 9A–D).

## Discussion

In this study, we investigated the relationship between IS and FGF23 in LVH. We found that IS increased levels of *FGF23* mRNA and markers of hypertrophy in cardiomyocytes. These data suggest that IS leads to LVH by increasing FGF23 expression.

IS is an endogenous agonist for AhR, (26) which is required for *GALNT3* gene expression. GALNT3 inhibits furin proprotein convertase processing by *O*-glycosylation of tyrosine at residue 178 of FGF23, which suppresses degradation of FGF23 and increases intact FGF23 (25, 27). Our study showed that IS upregulated GALNT3 and FGF23, and that this effect could be controlled by suppressing AhR (Figures 2, 7), supporting the above-mentioned pathways. Although FGF23 expression increased with IS treatment, furin was also mildly upregulated by IS. This finding suggests that FGF23 is likely to be upregulated by the GALNT3 pathway, not by a reduction in furin, and that increased expression of FGF23 may upregulate furin for negative feedback (Figures 3E, F). IS treatment also accelerated FGFR4 phosphorylation and induced cardiomyocyte hypertrophy.

Furthermore, we demonstrated that IS increased intact FGF23 protein expression in the heart, but not in the serum (Table 1 and Figure 5), suggesting that the effect of IS on FGF23 may be limited to organs such as the heart. A recent report also showed that FGF23 expression in the heart was associated with LVH using autopsy samples collected from patients with CKD (28). Additionally, rats that undergo nephrectomy show FGF23 expression in the heart (18). This evidence supports the notion that FGF23 expression in the heart is induced by the uremic condition containing higher serum IS concentrations.

**FIGURE 10:**
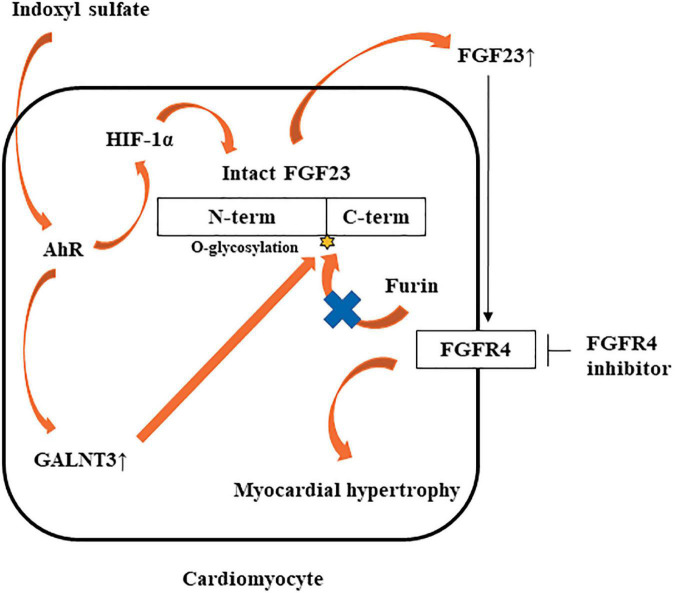
Potential mechanism by which IS may induce LVH *via* the FGF23-FGFR4 pathway. IS upregulates *FGF23* mRNA *via* AhR and HIF1α. IS also increases *GALNT3* gene expression, preventing furin-mediated degradation of intact FGF23. Increased FGF23 induces myocardial hypertrophy by FGFR4-dependent activation.

In this study, IS increased not only FGF23 protein expression, but also *FGF23* mRNA expression. GALNT3 induced by IS inhibited the degradation of FGF23 protein expression. Therefore, another mechanism was involved in increased *FGF23* mRNA induced by IS. Recent studies showed that inflammatory cytokines were direct regulators of FGF23 production in cardiac fibroblasts (29) and osteoblasts (24, 30). The induction of HIF1α in osteoblasts and osteocytes, in response to iron deficiency or hypoxia, increases FGF23 production (31). IL-1β significantly increases *FGF23* mRNA expression through a HIF1α-dependent mechanism (24). Therefore, some inflammatory cytokines induce *FGF23* mRNA *via* HIF1α. In this study, IS increased HIF1α *via* the IS-AhR pathway (Figures 7D, E), which suggested that the increase in *FGF23* mRNA induced by IS was partly mediated by HIF1α. On the basis of these findings, we propose a novel hypothesis on the role of the IS-FGF23-FGFR4 signaling pathway in uremic cardiomyopathy (Figure 10).

FGFR4 is one of four family members harboring tyrosine kinase domains and is most likely expressed only in cardiomyocytes and not in other cardiac cell types (17). Three FGF19 subfamily members (FGF19, FGF21, and FGF23) have the potential to bind FGFR4. H3B-6527 is a selective FGFR4 inhibitor (32). Faul et al. (33) showed that FGF23 caused LVH by FGFR-dependent activation of the calcineurin-NFAT signaling cascade, but it did not require klotho as a coreceptor. FGFR inhibition can reduce LVH caused by FGF23 independent of blood pressure. In our study, IS increased FGFR4 phosphorylation, and FGFR4 inhibition prevented IS-induced LVH, which suggested that IS affected the FGF23-FGFR4 signaling pathway in cardiomyocytes.

A previous report showed that active vitamin D inhibited FGF23-FGFR4 signaling and hypertrophy (18). Paracrine FGF23 activates FGFR tyrosine kinase, and induces activation of the renin–angiotensin system-mitogen-activated protein kinase, phospholipase C-gamma, phosphatidylinositol 3-kinase-AKT, and STAT pathways (34). Active vitamin D also inhibits expression of renin–angiotensin system-associated gene expression and cardiac fibrosis (35). Therefore, active vitamin D may have a role in preventing LVH. However, in our study, IS did not decrease active vitamin D in H9c2 cells (data not shown).

We used the LVH model with heminephrectomy in this study because this model shows mild CKD. Therefore, the elevation in serum FGF23 concentrations is not extremely high. Consequently, we could investigate the IS-FGF23 pathway without the confounding effect of other uremic toxins in this model. In sham mice, serum intact FGF23 concentrations were lower than the other groups. The sham mice were fed a normal phosphorus diet and experienced less stress than the other mice treated by a micro-osmotic pump (Table 1).

Previous studies have shown that FGF23 promotes myocardial fibrosis (36, 37). We also evaluated cardiac fibrosis in the mice in this study, but we found no significant difference among the groups (data not shown). The mouse model in this study is different from that used in previous studies (36, 37). Therefore, we speculate that cardiac fibrosis may occur later than cardiac hypertrophy or *via* another pathway in our model. A possibility is that β-catenin or the local renin–angiotensin system may induce cardiac fibrosis associated with FGF23 (36, 37).

In summary, this study shows that IS induces LVH *via* AhR and the FGF23-FGFR4 signaling pathway *in vitro* and *in vivo*. IS also upregulates GALNT3 and HIF1α through AhR, leading to the stabilization of FGF23. These data suggest that blocking the IS-AhR-FGF23-FGFR4 pathway is a new strategy to prevent LVH in patients with CKD.

## Data availability statement

The data that support the findings of this study are available from the corresponding author upon reasonable request.

## Ethics statement

The animal study was reviewed and approved by the Committee on Ethics of Animal Experimentation at Kyushu University Graduate School of Medical Sciences.

## Author contributions

HK performed most of the experiments and drafted the manuscript. TN was responsible for the experimental design, funding of the study, data interpretation, and manuscript development. KT supervised the cell culture experiments and animal experiments. MTo provided advice on the cell culture and animal experiments and contributed to revision of the manuscript. YU assisted with the experiments and data acquisition. SY and MTa contributed to revision of the manuscript. TK contributed to critical revision of the manuscript and supervision of the study. All authors provided critical reviews of the draft and approved the final version.

## Supplementary material

Supplementary Figure 1 IS induces hypertrophy *in vitro*. H9c2 cells were cultured with 0 or 1 mM IS for 72 h. Cell surface changes were measured using ImageJ software (National Institutes of Health, 165 Bethesda, MD) (*n* = 10). Student’s *t*-test was used for analysis. **P* < 0.01 versus control.

Supplementary Figure 2 IS increases mRNA levels of markers of hypertrophy and fibrosis in medium with 4% albumin. H9c2 cells were cultured with 0, 0.25, or 1.0 mM IS in medium with 4% albumin for 24 h, and mRNA expression levels were analyzed by real-time PCR. **(a)** *ANF*, **(b)** *BNP*, **(c)** *beta MHC*, **(d)** *alpha SMA*, **(e)** collagen I, and **(f)** *FGF23* mRNA levels were significantly increased by IS (*n* = 6). Data were analyzed by one-way analysis of variance. **P* < 0.01, ^**^*P* < 0.05.

Supplementary Figure 3 IS increases FGF23 protein expression and FGFR4 phosphorylation in medium with 4% albumin. H9c2 cells were cultured with 0, 0.25, or 1.0 mM IS in medium with 4% albumin for 48 h. Alpha, beta-tubulin protein expression was examined as an internal control (*n* = 6). Data were analyzed by one-way analysis of variance. **P* < 0.01, ^**^*P* < 0.05 versus IS 0 mM. **(a,b)** Western blotting of FGF23 protein. **(c,d)** Western blotting of FGFR4 protein expression and FGFR4 phosphorylation.
