# Supplementary figures and images for: Indoxyl sulfate induces left ventricular hypertrophy via the AhR-FGF23-FGFR4 signaling pathway
Source: Front Cardiovasc Med. 2023 Feb 21;10:990422. doi: 10.3389/fcvm.2023.990422 (PMC9988908; doi:10.3389/fcvm.2023.990422)

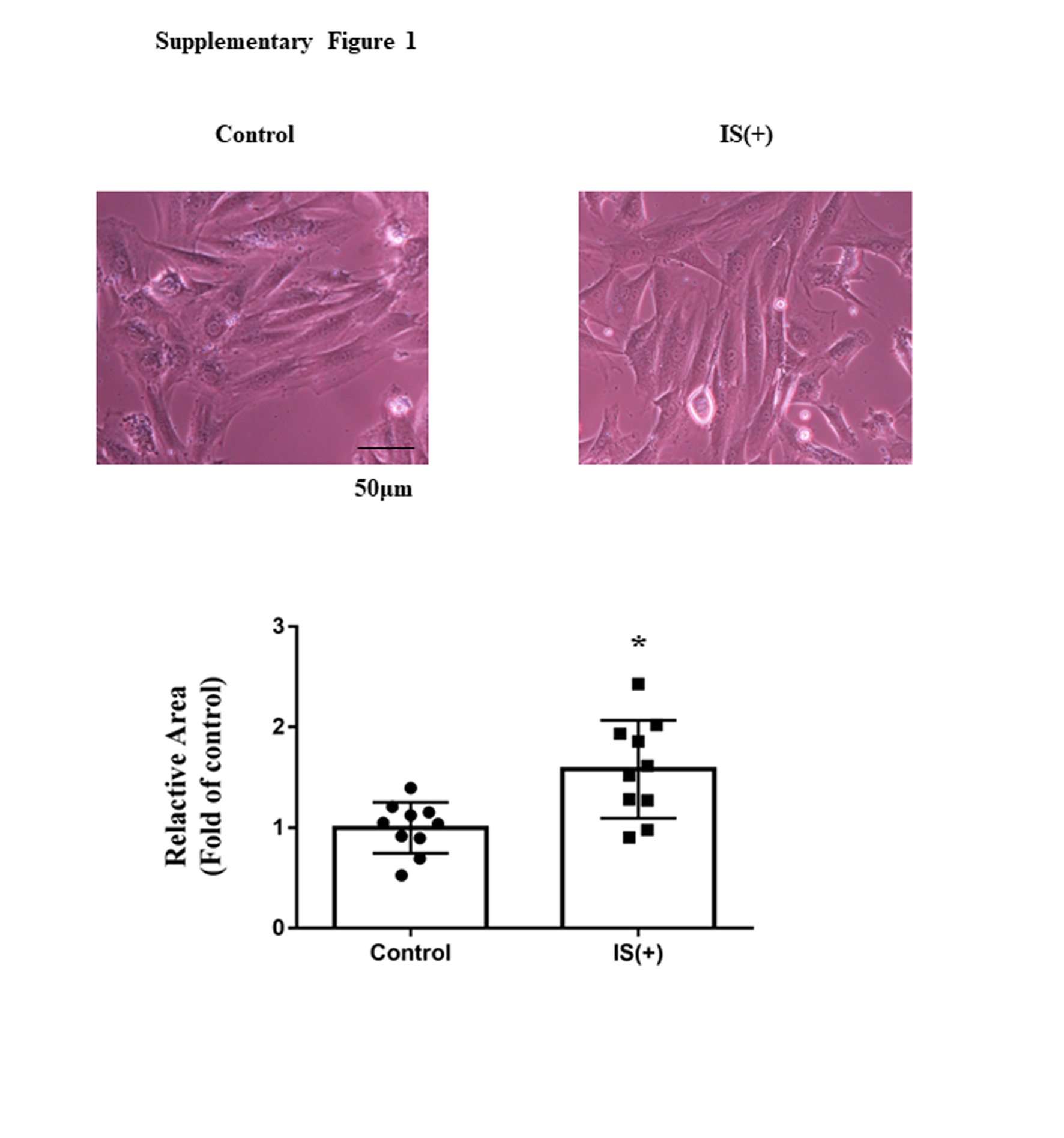

Supplement: Supplementary Figure 1 — IS induces hypertrophy in vitro. H9c2 cells were cultured with 0 or 1 mM IS for 72 h. Cell surface changes were measured using ImageJ software (National Institutes of Health, 165 Bethesda, MD) (n = 10). Student’s t-test was used for analysis. *P < 0.01 versus control. [file Image_1.TIF]

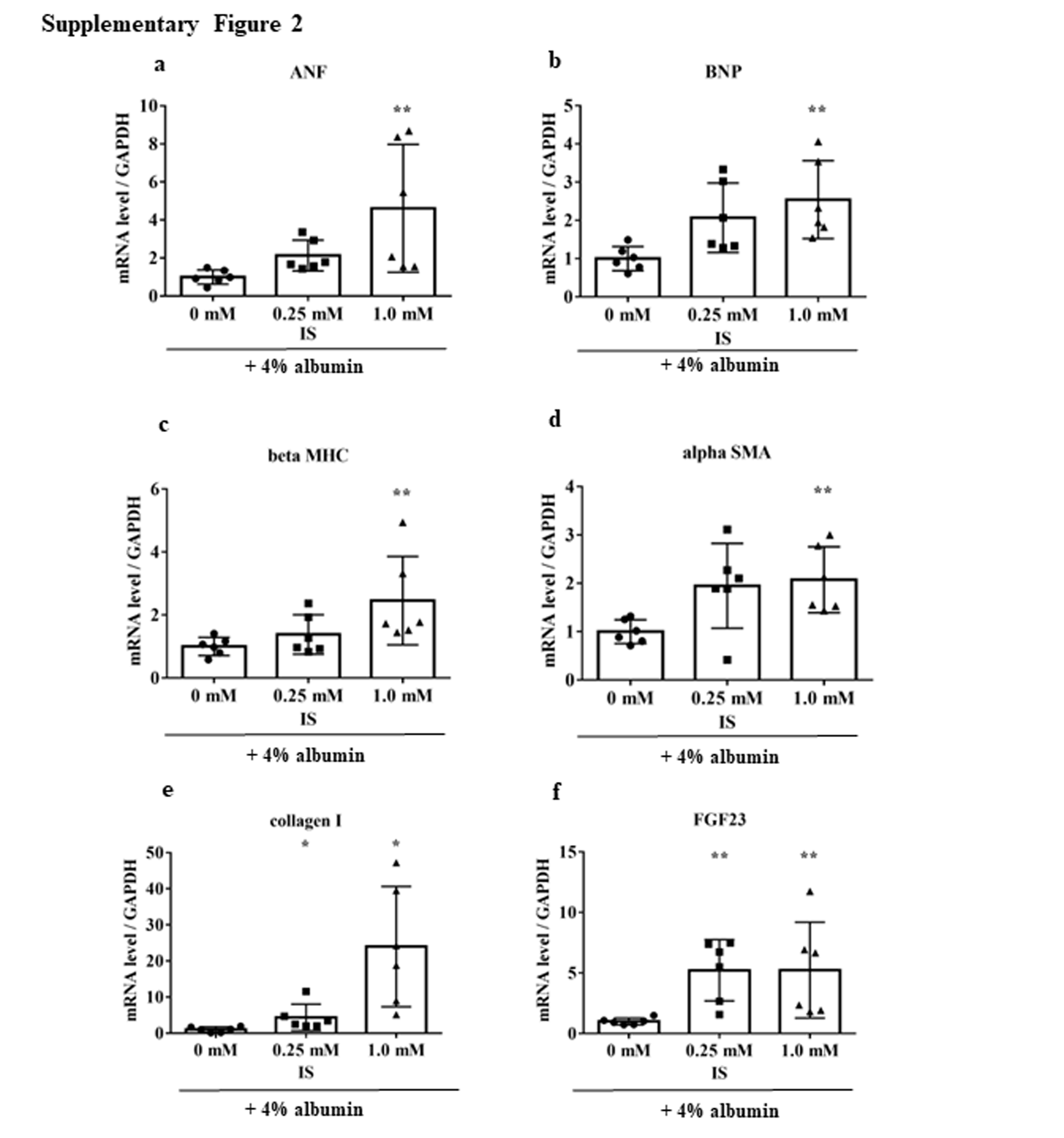

Supplement: Supplementary Figure 2 — IS increases mRNA levels of markers of hypertrophy and fibrosis in medium with 4% albumin. H9c2 cells were cultured with 0, 0.25, or 1.0 mM IS in medium with 4% albumin for 24 h, and mRNA expression levels were analyzed by real-time PCR. (a) ANF, (b) BNP, (c) beta MHC, (d) alpha SMA, (e) collagen I, and (f) FGF23 mRNA levels were significantly increased by IS (n = 6). Data were analyzed by one-way analysis of variance. *P < 0.01, **P < 0.05. [file Image_2.TIF]

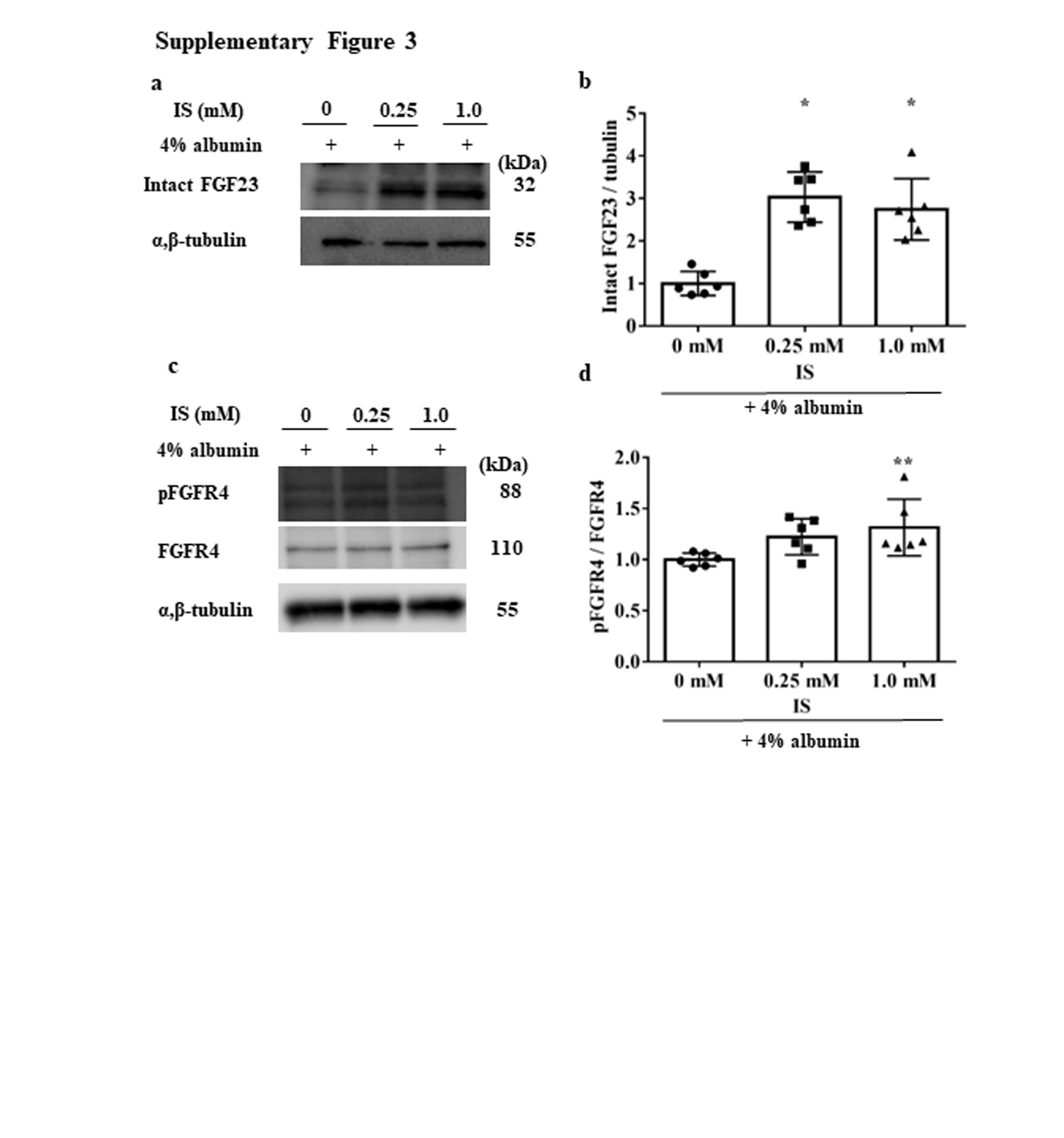

Supplement: Supplementary Figure 3 — IS increases FGF23 protein expression and FGFR4 phosphorylation in medium with 4% albumin. H9c2 cells were cultured with 0, 0.25, or 1.0 mM IS in medium with 4% albumin for 48 h. Alpha, beta-tubulin protein expression was examined as an internal control (n = 6). Data were analyzed by one-way analysis of variance. *P < 0.01, **P < 0.05 versus IS 0 mM. (a,b) Western blotting of FGF23 protein. (c,d) Western blotting of FGFR4 protein expression and FGFR4 phosphorylation. [file Image_3.TIF]
